# Phenotypic Changes in Transgenic Tobacco Plants Overexpressing Vacuole-Targeted *Thermotoga maritima* BglB Related to Elevated Levels of Liberated Hormones

**DOI:** 10.3389/fbioe.2015.00181

**Published:** 2015-11-09

**Authors:** Quynh Anh Nguyen, Dae-Seok Lee, Jakyun Jung, Hyeun-Jong Bae

**Affiliations:** ^1^Department of Bioenergy Science and Technology, Chonnam National University, Gwangju, South Korea; ^2^Bio-Energy Research Center, Chonnam National University, Gwangju, South Korea

**Keywords:** *Thermotoga maritima*, hyperthermostable β-glucosidase BglB, C-terminal AFVY tetrapeptide, vacuole-targeted, hormone conjugates, shoot apical meristem

## Abstract

The hyperthermostable β-glucosidase *BglB* of *Thermotoga maritima* was modified by adding a short C-terminal tetrapeptide (AFVY, which transports phaseolin to the vacuole, to its C-terminal sequence). The modified β-glucosidase *BglB* was transformed into tobacco (*Nicotiana tabacum* L.) plants. We observed a range of significant phenotypic changes in the transgenic plants compared to the wild-type (WT) plants. The transgenic plants had faster stem growth, earlier flowering, enhanced root systems development, an increased biomass biosynthesis rate, and higher salt stress tolerance in young plants compared to WT. In addition, programed cell death was enhanced in mature plants. Furthermore, the C-terminal AFVY tetrapeptide efficiently sorted *T. maritima* BglB into the vacuole, which was maintained in an active form and could perform its glycoside hydrolysis function on hormone conjugates, leading to elevated hormone [abscisic acid (ABA), indole 3-acetic acid (IAA), and cytokinin] levels that likely contributed to the phenotypic changes in the transgenic plants. The elevation of cytokinin led to upregulation of the transcription factor *WUSCHELL*, a homeodomain factor that regulates the development, division, and reproduction of stem cells in the shoot apical meristems. Elevation of IAA led to enhanced root development, and the elevation of ABA contributed to enhanced tolerance to salt stress and programed cell death. These results suggest that overexpressing vacuole-targeted *T. maritima* BglB may have several advantages for molecular farming technology to improve multiple targets, including enhanced production of the β-glucosidase BglB, increased biomass, and shortened developmental stages, that could play pivotal roles in bioenergy and biofuel production.

## Introduction

β-glucosidase is critical for many developmental processes in plants, and the hydrolysis of phytohormone conjugates is one of its most important roles (Schliemann, 1984; Sembdner et al., 1994; Kleczkowski et al., 1995). The rolC gene of the bacterial pathogen *Agrobacterium rhizogenes* encodes β-glucosidase, and results in abnormal development when transformed into plants. In particular, heterologous β-glucosidase can release active forms of phytohormones from their inactive conjugates that consist of glycoside links (Spena et al., 1992; Brzobohaty et al., 1993). Inactive conjugates of each phytohormone can be found abundantly in plant tissues. Their active forms are liberated via β-glucosidase-mediated hydrolysis. Furthermore, many studies have revealed that the inactive forms of phytohormone conjugates act as reversible deactivated storage molecules, and are important for the regulation of physiologically active hormone levels; however, their normal biological functions remain unknown (Staswick, 2009; Piotrowska and Bajguz, 2011).

The vacuole is considered a storage organelle and is an important component of the secretory pathway in plants. Detailed knowledge of the sorting mechanisms, out of and into the vacuole, is lacking (Hall, 2000; Vitale and Hinz, 2005). Previous studies of several lytic enzymes that are specifically targeted to the vacuole (e.g., phaseolin) have revealed some sorting signals (N-terminal or C-terminal polypeptides or internal sequences) that can sort proteins into the vacuole (Frigerio et al., 2001; De Marcos Lousa et al., 2012). Unfortunately, because the internal environment of the vacuole leads to the rapid degradation and hydrolysis of proteins, and other compounds, it has been difficult to determine whether heterologous expressed proteins maintain their functions and features inside the vacuole.

We previously expressed the hyperthermostable β-glucosidase BglB of *T. maritima* in tobacco plants to obtain transgenic plants for application in bioconversion. The optimal temperature and pH of the plant-expressed BglB were 80°C and 4.5, respectively (Jung et al., 2010). Moreover, we also observed some phenotypic modifications, such as longer stems, larger leaves, and shortened developmental stages (Jung et al., 2010, 2013), which we hypothesized may have been due to changes in hormone homeostasis. Therefore, in the present study, we overexpressed heterologous BglB of *T. maritima* in tobacco plants. We targeted the vacuole by insertion of the AFVY tetrapeptide to examine whether BglB maintain its functions of hydrolyzing glycoside bonds to release free hormones from its conjugates, and to determine how such changes in hormones levels may affect the growth and development of transgenic plants. All of the changes in the aboveground or belowground organs in plants can be explained via the development, division, and reproduction of stem cells harbored in the shoot and root apical meristems, which are regulated by the expression of homeodomain genes and hormone levels. For example, in the shoot apical meristem, the transcription factor *WUSCHELL (WUS)* can be upregulated via cytokinin, and a group of dividing cells called the quiescent center (QC) is upregulated by indole 3-acetic acid (IAA) in the root apical meristem (Kerk et al., 2000; Overvoorde et al., 2010; Yadav et al., 2010; Zhao et al., 2010).

## Materials and Methods

### Vector Constructions, Plant Transformation, and Molecular Analysis

**Figure 1:**
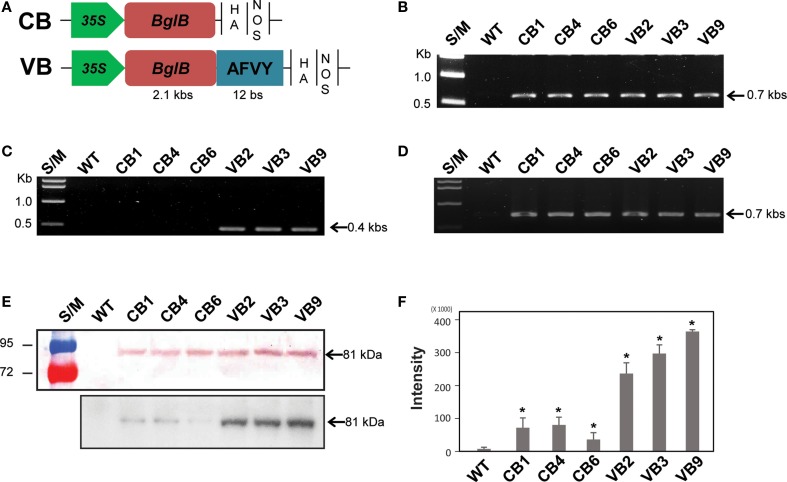
**Schematic representation of the BglB expression cassettes and confirmation of heterologously expressed BglB in the transgenic plants**. **(A)** Vector constructions of CB (cytosol-targeted BglB) and VB (vacuole-targeted BglB, with the 12 bp represent AFVY tetrapeptide). **(B)** Confirmation of the presence of BglB in CB and VB constructs in tobacco genome by PCR genomic DNA. **(C)** Confirmation of the presence of the sequence encoding AFVY tetrapeptide in the C-terminal of BglB of VB transformants by PCR genomic DNA. **(D)** Confirmation of the transcript sequence encoding BglB in CB and VB constructs after mRNA extraction, cDNA synthesis, and RT-PCR. **(E)** Confirmation of the presence of heterologously expressed BglB in the transgenic plants, with BglB present in total soluble protein (TSP) extracted from leaf of CB and VB transgenic plants but no from WT plant (above panel, with 10 μg of TSP was used); and the presence of the heterologous BglB in TSP extracted from isolated vacuole of CB and VB plants but no from those of WT plant (below panel, with 10 μg of TSP from isolated vacuole was used), after concentrated by UFC 710008/Centricon Plus-70 Centrifugal Filter (EM Millipore, USA) by using fluorophore-conjugated secondary antibody anti-rabbit IgG, incubate with substrate within 5 min before transferring to exposure film and keeping within 10 min. **(F)** The intensity of bands exposed in below panel from **(E)**, measured by Adobe Photoshop CS6. *indicates significant differences from the control (WT) (*P* < 0.05).

For cytosol expression, the full-length sequence of the *T. maritima* β-glucosidase *BglB* gene (Jung et al., 2010) was constructed under control of the 35S promoter, and named Cyt-BglB (CB). For vacuole targeting, *BglB* was modified by replacing its stop codon with nucleotide sequences encoding the AFVY signal tetrapeptide from the vacuolar storage glycoprotein phaseolin (Frigerio et al., 2001), with a stop codon inserted at the end, and named Vac-BglB (VB). According to previous studies, AFVY tetrapeptide signals are sufficient to target a heterologous protein to the vacuole (Frigerio et al., 2001; Lau et al., 2010). The 35S promoter was also used for vacuole targeting of the recombinant variants. These expression cassettes were then sub-cloned into the modified multiple cloning sites of the binary vector pCambia 2300 (Kim et al., 2010), as shown in Figure 1A. *Agrobacterium tumefaciens* strain GV3013 was used for transformation of tobacco (*Nicotiana tabacum* L.) via the leaf-disk method (Helmer et al., 1984). Transformed shoots were selected on solid Murashige–Skoog (MS) medium (Murashige and Skoog, 1962) containing 100 μg/ml kanamycin and 500 μg/ml cefotaxime. Transgenic tobacco plants were grown in a growth chamber under a 16-/8-h light/dark cycle at 25 ± 3°C. After the presence of the transgene was confirmed by genomic DNA polymerase chain reaction (PCR), reverse transcription (RT)-PCR, and Western blotting, the T_0_ generation of transgenic and wild-type (WT) plants was moved to a greenhouse for development.

Total genomic DNA was isolated from the T_0_ generation transgenic plant leaves using genomic DNA extraction buffer [200 mM Tris–HCl, 250 mM NaCl, 25 mM Na_2_-EDTA, 0.5% sodeum dodecyl sulfate (SDS)]. The concentration of genomic DNA was measured using a NanoDrop spectrophotometer (Thermo Fisher Scientific, USA). To confirm the presence of *BglB*, PCR of genomic DNA was performed using two sets of flanking primers: first FP 5′-GTC GCT CAT CAC GAA ACC GT-3′ and RP 5′-ACT ACA GAG GAA AAG GTG AA-3′ for checking the presence of a 0.7-kb sequence within *BglB* in the CB and VB constructs, and second FP 5′-TAT GCA GGC TCC CAC CCC TT-3′ and RP 5′-GTA TAC GAA TGC ACT ACA GA-3′ for checking the presence of a 0.4-kb sequence within *BglB* and nucleotides sequences of AFVY tetrapeptide in VB constructs. For RT-PCR, total RNA was extracted from the leaf tissues and cDNA was synthesized using avian myeloblastosis virus (AMV) reverse transcriptase (Promega, USA) with random hexamers, and the RT-PCR of *BglB* was performed using first primers set mentioned above. RT-PCR was also performed to determine the expression level of *N. tabacum WUS* (JQ686923.1) after RNA was extracted from the stems of seedlings and cDNA was synthesized, with the specific primers: FP 5′-ATG CAC ATG AGA GGT GTT TG-3′ and RP 5′-TTA AGG GGA ATT AGG AGA TC-3′.

BglB proteins were extracted from the T_0_ to T_3_ generation transgenic tobacco leaves by grinding the leaf material to a powder in liquid nitrogen and then suspending the powder in protein extraction buffer at pH 8.0 (50 mM Tris–HCl, 5 mM Na_2_-EDTA, 20 mM Na_2_S_2_O_5×_5H_2_O, 100 mM KCl, 5% glycerol, 1% β-mercaptoethanol). Leaf debris was removed by centrifugation at 13,000 ×* g* for 20 min at 4°C. Total soluble protein (TSP) in the supernatants was measured using the Bradford method (Bradford, 1976). Using transfer buffer (39 mM glycine, 48 mM Tris, 10% SDS, 20% methanol), 10 μg of protein was electrophoresed on 12% polyacrylamide gels and transferred to polyvinylidene fluoride membranes (Immobilon-P; Millipore). The membrane was blocked by incubation with 5% skimmed milk (Difco, USA) in phosphate-buffered saline at pH 7.0 (1 mM KH_2_PO_4_, 10 mM Na_2_HPO_4_ × 12H_2_O, 137 mM NaCl, 2.7 mM KCl), and then incubated with a polyclonal anti-β-glucosidase antibody as the primary antibody. Alkaline phosphatase-conjugated goat anti-rabbit IgG antibody (Promega, USA) was used at a 1:2500 dilution as the secondary antibody. For detecting β-glucosidase *BglB* targeting vacuole, total protein from isolated vacuole was used to conduct western blot with the same as above, except incubating with a fluorophore-conjugated secondary antibody anti-rabbit IgG (H + L) (DyLight TM 680 Conjugate) (red) in a 1:2500 dilution.

### Growth Conditions, Sampling, and Phenotypic Observation

After the presence of the transgene was confirmed, T_0_ generation transgenic and WT plants were moved to the greenhouse for development. Seeds from the T_0_ generation transgenic plants were sprayed in MS medium containing kanamycin and grown in a growth chamber under a 16-/8-h light/dark cycle at 25 ± 3°C to produce the T_1_ generation, and the germination day was recorded. After 2 weeks, seedlings from ten lines of the CB and VB transgenic plants were used to determine β-glucosidase activity. A 100 mg sample of grinded powder from the leaf was used to extract TSP (Jung et al., 2010), and after checking TSP by the Bradford method, an amount of extracted protein equivalent to 10 μg of TSP were used to examine β-glucosidase enzymatic activity by using p-nitrophenyl b-d-glucopyranoside (pNPG) as the substrate. One unit of β-glucosidase is defined as the amount of enzyme that released 1 mmol of p-nitrophenol from the pNPG substrate under the assay conditions described below. The assay mixture containing 10 mM pNPG in citrate–phosphate buffer (pH 4.5) was incubated with the enzyme for 30 min at 70°C in a total volume of 1 ml. The reaction was stopped by adding 1M Na_2_CO_3_, and absorbance was measured at 405 nm. Based on these results, we selected three transgenic lines from each of the CB and VB transgenic plants that showed the highest crude extract β-glucosidase enzymatic activity. Thirty plants from each of the chosen lines were grown in a growth chamber and then moved to 10-l pots in soil–perlite mixtures at 25 ± 3°C under a 16-/8-h light/dark photoperiod and a light intensity of 100 mmol m^–2^ s^–1^ in a greenhouse for further analysis.

The fifth leaf from the tops of three plants of each of the chosen transgenic lines and W plants was harvested at the same time, and after each 20 days, from 30 to 90 day after germination (DAG), stored at −70°C and ground in liquid N_2_ to analyze β-glucosidase enzymatic activity.

The phenotypic characteristic of the transgenic and WT plants were also recorded from the T_1_ to T_3_ generation of transgenic plants, including: stem height, number of leaves, root lengths, number of lateral roots, time from germination to initial flowering, and dry weight (the leaves, stems, and roots of non-sample plants were separately harvested and freeze-dried after harvesting the seeds). The carbohydrate content of each part of the plant was also determined using gas chromatography (Coleman et al., 2009).

To conduct a salt stress tolerance experiment in the growth chamber, after germination, the seedlings were transferred to new MS media (without sucrose) containing 200 mM NaCl, and phenotypic characteristics were measured after 15 DAG. Mature (80 DAG) transgenic and WT plants grown in the greenhouse were used for the salt stress experiment, which the plants were watered with the same amount of 200 mM NaCl within 10 days. The weight of spots (including spot, soil, and plants) was measured before and after 10 days NaCl treatment. Simultaneously, the same position on the leaf (10th leaf from the ground to above) was harvested, ground with liquid N_2_, chlorophyll was extracted 90% ethanol, boiled for 5 min and absorbance was measured at an optical density of 620 nm to calculate the chlorophyll concentration (Lichtenthaler and Wellburn, 1983).

### Phytohormone Extraction and Measurement

The phytohormones [include abscisic acid (ABA), IAA, and cytokinin] from young leaves or seedlings of CB, VB, and WT plants were extracted with 80% methanol (Oliver et al., 2007) and measured using the Phytodetek competitive enzyme-linked immunosorbent assay (ELISA) kit (Agdia; Elkhardt, IN, USA) the Phytodetek competitive ELISA kits (Agdia). Briefly, young leaves or seedlings of transgenic and WT plants were harvested and ground in N_2_ liquid and stored at −70°C for further analysis. One gram of ground powder was mixed with 1 ml 80% methanol and incubated overnight at −4°C. One milliliter (ml) of the supernatant was collected after centrifuging at 13,000 rpm for 10 min to remove debris, and then freeze-dried. The freeze-dried powder was used to measure the levels of each hormone, according to the kit protocols. Each measurement was conducted in triplicate.

### Vacuoles Isolation

For purification of vacuole-targeted BglB, transformed protoplasts from young plants (30 DAG) were isolated by hydrolysis with cell-wall hydrolysis enzymes and fractionated by ultracentrifugation according to Mettler et al. (Mettler and Leonard, 1979) and Raikhel et al. (Robert et al., 2007), with some modifications. Due to the requirement of a highly purified of vacuole, the transformed protoplasts were loaded on top of step gradients consisting of 4, 7, 12, and 15% Ficoll, and centrifuged at 97,000 ×* g* for 4 h. The vacuole was isolated in the top layer of the fraction, and then disrupted by sonication before measuring β-glucosidase enzymatic activity with 10 μg TSP.

## Results

### β-glucosidase Enzymatic Activity from Isolated Vacuoles and Total Hormone Levels were Significantly Higher in Transgenic than in WT Plants

Hormone conjugates, which are found in each class of plant hormones, are mainly localized in the vacuoles of plant. The mechanism controlling their transport across membranes and between plant organs remain unknown (Bajguz and Piotrowska, 2009). To analyze the effects of thermostable *T. maritima* BglB on changes in phytohormone metabolism and the consequences for plant development, we built two constructs of BglB. The CB construct was for ectopic expression of *BglB* in the cytosol, and the VB construct was for expression of vacuole-targeted BglB, under the control of the 35S promoter (Figure 1A). In total, 10 and 12 lines of CB and VB transgenic plants, respectively, were confirmed, and three of the lines were used for further analysis after confirmation of the transgenes by genomic DNA PCR, reverse transcription (RT)-PCR, and Western blotting (Figures 1B–E). The presence of the nucleotide sequences encoding the AFVY tetrapeptide in the VB construct was confirmed using PCR with a reverse primer specific to the VB construct (Figure 1C), and the transcript of the heterologous BglB in the transgenic plants were confirmed by RT-PCR (Figure 1D). The existence of BglB in TSP from the CB and VB plants had molecular weights similar to BglB, as mentioned in the previous study (Jung et al., 2010), were detected by western blot (Figure 1E, above panel), presented no different between CB and VB plants, but showed significantly higher level of BglB in the isolated vacuoles of VB plant compared to CB plant (Figure 1E, below panel), indicated by the higher intensity of the BglB band exposed by the present of BglB in the isolated vacuoles from the VB transgenic than from the CB transgenic and WT plants (Figure 1F). These results obviously indicate that VB plants were the highest vacuole-targeted heterologous BglB.

**Figure 2:**
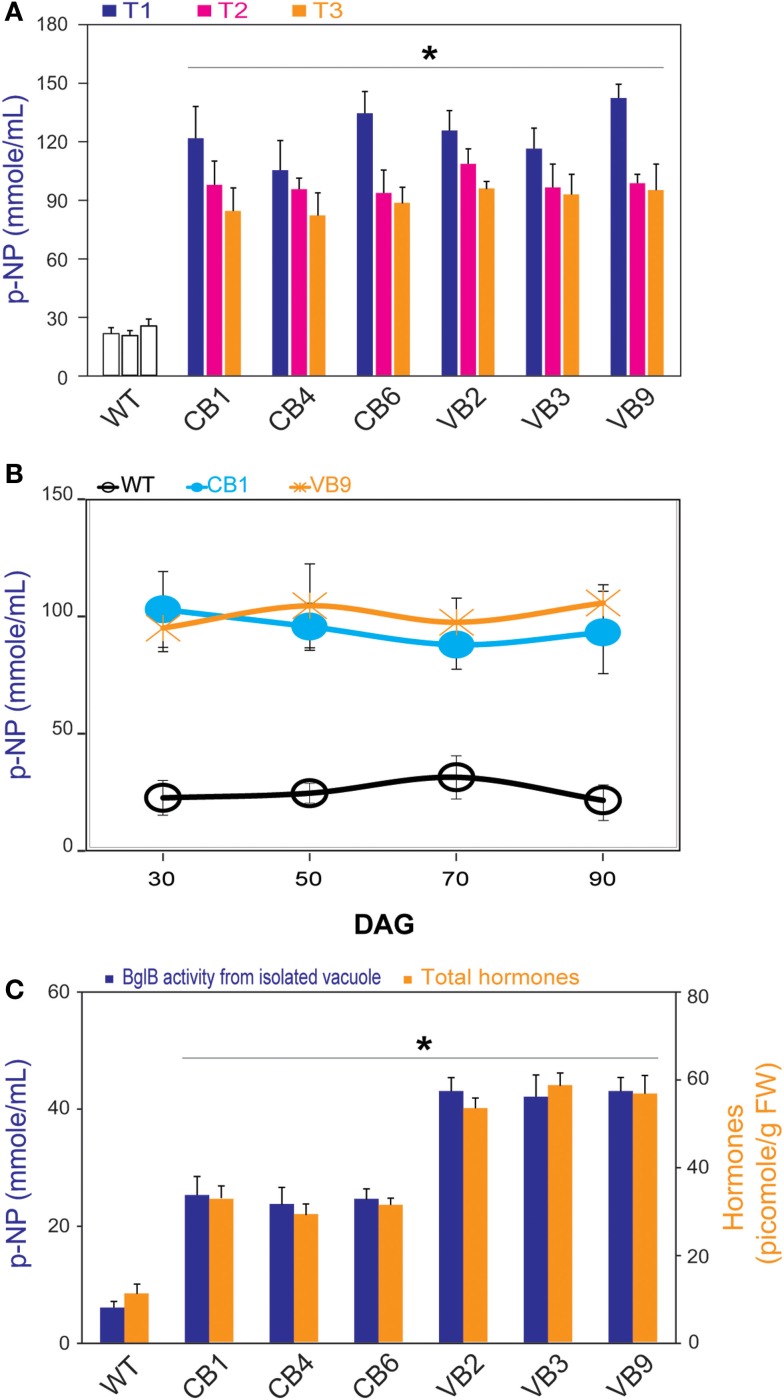
**β-glucosidase enzymatic activity and total hormones levels**. **(A)** Significant higher β-glucosidase enzymatic activity of heterologously expressed BglB during three generations of CB and VB transgenic plants compared to WT plant. **(B)** Profile pattern of β-glucosidase enzymatic activity of heterologously expressed BglB in CB and VB transgenic plants in T_1_ generations, from 30 to 90 DAG revealed the significant higher β-glucosidase enzymatic activity of CB and VB transgenic plants compared to WT plants. **(C)** Significant higher β-glucosidase enzymatic activity of heterologously expressed BglB from isolated vacuole and the total hormones levels of CB and VB transgenic plants compared to WT plant at 14 DAG. Vacuole was isolated after protoplasts preparation from 14 DAG tobacco seedlings (WT and transgenic), broken by sonication within 5 min in 0.5 cycle/60% amplitude in protein extraction buffer at pH 8.0 (50 mM Tris–HCl, 5 mM Na_2_-EDTA, 20 mM Na_2_S_2_O_5_ × 5H_2_O, 100 mM KCl, 5% glycerol, 1% β-mercaptoethanol), and concentrated by UFC 710008/Centricon Plus-70 Centrifugal filter (EM Millipore, USA). Average values were calculated from triplicate (*n* = 3) of each transgenic lines and WT plants. *indicates significant differences from the control (WT) (*P* < 0.05).

The three best-performing T_0_ generation transgenic lines were selected according to their β-glucosidase enzymatic activity, and then self-pollinated. The β-glucosidase enzymatic activity was significantly higher in the transgenic plants, compared to WT plants over three generations (T_1_ to T_3_; Figure 2A). A slight reduction of β-glucosidase enzymatic activity after a few generations was observed, possibly due to factors such as epigenetic silencing mechanisms (Iyer et al., 2000; Matzke et al., 2000). Heterologous *BglB* was also stably expressed in the transgenic plants, as indicated by the pattern of β-glucosidase enzymatic activity during plant development from 30 to 90 days after germination (DAG) of the CB1 and VB9 transgenic plants (Figure 2B).

To examine the efficiency of vacuole targeting, we isolated the vacuoles of the WT plants, and the CB and VB transgenic lines, from the T_1_ generation. The results showed that the isolated vacuoles of the VB transgenic lines had the highest β-glucosidase enzymatic activity, compared to Cyt-BglB and WT plants (Figure 2C). In particular, compared to WT plants, increased β-glucosidase enzymatic activity of 452 and 759% were recorded in the vacuoles of CB1 and VB2 transformants, respectively. These results accompany to above identification (presented in Figure 1E), indicate that the VB constructs which imposed AFVY tetrapeptide effectively targeted β-glucosidase to the vacuole, and that BglB was still active in the vacuole.

Moreover, significantly higher total hormone (including IAA, ABA, and cytokinin) levels were recorded in the transgenic plants compared to the WT plants, based on the ELISA results, with the highest hormone levels obtained from the VB transformants (Figure 2C). In particularly, maximum increases of 268 and 463%, when comparing total extracted hormone levels in CB1 and VB3 to WT plants, respectively, were attributed to higher levels of each hormone in the transgenic plants (Figure S1 in Supplementary Material).

### Pronounced Phenotypic Changes in the Transgenic Plants

**Figure 3:**
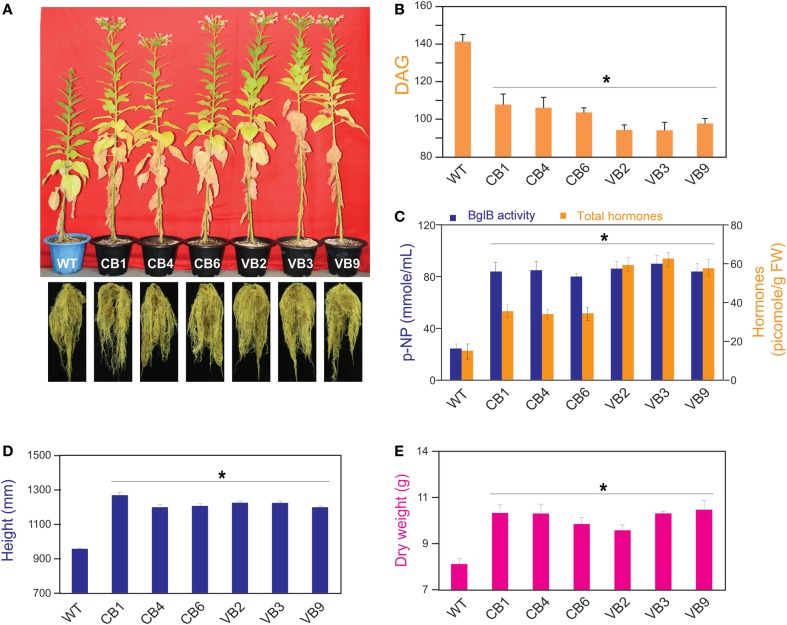
**Pronounced phenotypic changes occurred in heterologously expressed BglB transgenic tobacco plants**. **(A)** Faster growth in stem height, shortened growth cycle with earlier flowering, and enhanced root development in the transgenic plants compared to WT plants. **(B)** Time required from germination to flowering of the transgenic vs. WT plants, showed the shortest time required belong to VB plants. **(C)** Combination of β-glucosidase enzymatic activity and total hormones levels of the transgenic and WT plants, measured by harvesting the fifth leaf from the top of plant immediately after flowering. **(D)** Stem height and **(E)** dry weight of the transgenic and WT plants were calculated after harvesting seeds and freeze-dried. Average values were calculated from data recorded from twenty individuals (*n* = 20) of each transgenic lines and WT plants for **(B,D,E)**, and from triplicate (*n* = 3) of each transgenic lines and WT plants for **(C)**. *indicates significant differences from the control (WT) (*P* < 0.05).

The transgenic CB and VB tobacco plants displayed pronounced phenotypic changes compared to WT plants. Phenotypic characteristics such as stem height, time from germination to initial flowering, and dry weight were proportional to the levels of β-glucosidase enzymatic activity of transgenic and WT plants, suggesting a correlation between the enhancement of β-glucosidase enzymatic activity and these phenotypic changes. In particular, faster development was observed in the transgenic plants than in the WT plants, as indicated by increased stem height, earlier flowering, increased biomass accumulation, and enhanced root system development (Figure 3A; Figure S2A in Supplementary Material). Moreover, a shorter time from germination to initial flowering was recorded in the T_1_ generation of transgenic plants compared to WT plants. We reported an average of 103.6 and 94.1 DAG in the CB and VB transgenic plants, respectively, compared to 141.7 DAG in WT plants; Figure 3B), and similar results were observed for the T_2_ and T_3_ generations (Figure S2B in Supplementary Material). Higher β-glucosidase enzymatic activity and total hormone levels were recorded at flowering time, with maximum increases in total hormone levels of 222 and 387% for CB1 and VB3 compared to WT plants, respectively (Figure 3C; Figure S2C in Supplementary Material), while no significant differences in β-glucosidase enzymatic activity between the CB and VB transgenic plants was observed (Figure 3C). These results indicate that more liberated hormones were released in the VB than the CB transgenic plants. After the seeds were harvested, the stem height and dry weight of total biomass accumulation were significantly higher in the mature transgenic plants than in the WT plants, with maximum increases of 133% for stem height (CB1 compared to WT plants) and 124% for total dry weight (VB9 compared to WT plants; Figures 3D,E). Similar results were obtained for the T_2_ and T_3_ generations (Figures S2D,E in Supplementary Material). These results clearly indicated that the increase in liberated hormone levels (particularly IAA and cytokinin) contributed to increased biomass accumulation, despite the shortened growth cycle (earlier flowering after germination) in the transgenic plants. The same phenotypic characteristics were observed in previous studies that targeted β-glucosidase to either general or particular cellular compartments (Jung et al., 2010; Jin et al., 2011). However, despite the significant changes in biomass accumulation and shortened growth cycle, no significant differences in carbohydrate content were observed between the transgenic and WT plants (Table 1), indicating that only total biomass accumulation was influenced in the transgenic plants.

**Table 1:** **Comparison of carbohydrate content in leaves, stem, and roots in WT and vacuole-targeted *T. maritima* BglB transgenic plants**.

	(%)	WT	CB1	CB4	CB6	VB2	VB3	VB9
Leaves	Rhamnose	2.3 ± 0.2	2.2 ± 0.3	2.3 ± 0.5	2.6 ± 0.6	2.4 ± 0.3	2.6 ± 0.4	2.6 ± 0.5
	Arabinose	1.4 ± 0.1	1.4 ± 0.2	1.5 ± 0.1	1.5 ± 0.3	1.1 ± 0.1	1.1 ± 0.1	1.1 ± 0.1
	Xylose	1.6 ± 0.2	1.7 ± 0.3	1.6 ± 0.2	1.7 ± 0.0	1.5 ± 0.1	1.6 ± 0.1	1.3 ± 0.0
	Mannose	0.7 ± 0.1	0.9 ± 0.1	1.0 ± 0.2	0.7 ± 0.1	0.7 ± 0.2	0.7 ± 0.3	1.1 ± 0.0
	Galactose	2.1 ± 0.2	2.2 ± 0.4	2.2 ± 0.4	2.2 ± 0.4	2.0 ± 0.3	2.2 ± 0.5	1.9 ± 0.1
	Glucose	45.3 ± 3.5	46.2 ± 4.1	44.6 ± 3.3	44.9 ± 0.8	46.1 ± 3.8	43.9 ± 2.1	45.1 ± 1.9
	Total	53.4 ± 2.8	54.6 ± 3.3	53.2 ± 4.2	53.6 ± 2.6	53.8 ± 4.1	52.1 ± 3.2	53.1 ± 2.5
Stems	Rhamnose	1.8 ± 0.4	1.2 ± 0.1	0.9 ± 0.4	1.2 ± 0.2	1.0 ± 0.2	0.8 ± 0.1	1.2 ± 0.2
	Arabinose	0.7 ± 0.2	0.8 ± 0.0	1.0 ± 0.0	1.1 ± 0.0	1.3 ± 0.0	0.9 ± 0.1	1.1 ± 0.1
	Xylose	5.6 ± 0.7	5.9 ± 1.5	6.2 ± 0.6	7.1 ±1.1	6.2 ± 0.1	5.8 ± 0.7	5.9 ± 0.9
	Mannose	1.0 ± 0.2	1.3 ± 0.3	1.1 ± 0.3	1.2 ± 0.0	1.1 ± 0.1	1.2 ± 0.1	1.2 ± 0.0
	Galactose	1.2 ± 0.1	1.4 ± 0.2	1.3 ± 0.2	1.2 ± 0.1	1.0 ± 0.1	1.1 ± 0.1	1.0 ± 0.2
	Glucose	44.7 ± 1.2	45.7 ± 6.1	46.2 ± 5.6	47.1 ± 5.4	46.1 ± 2.9	45.5 ± 3.6	43.9 ± 3.2
	Total	55.0 ± 4.2	56.3 ± 6.5	56.7 ± 7.2	58.9 ± 6.5	56.7 ± 3.1	55.3 ± 4.7	54.3 ± 4.8
Roots	Rhamnose	1.3 ± 0.5	1.1 ± 0.0	1.3 ± 0.3	1.0 ± 0.3	1.3 ± 0.1	1.4 ± 0.2	1.3 ± 0.3
	Arabinose	0.8 ± 0.1	0.9 ± 0.1	1.1 ± 0.0	0.8 ± 0.1	1.1 ± 0.1	0.8 ± 0.2	0.9 ± 0.2
	Xylose	6.4 ± 0.5	6.9 ± 1.1	7.1 ± 1.1	6.4 ± 0.3	6.8 ± 0.5	6.6 ± 0.4	6.7 ± 1.9
	Mannose	1.1 ± 0.5	1.3 ± 0.1	1.2 ± 0.0	1.3 ± 0.0	1.2 ± 0.1	1.2 ± 0.0	1.0 ± 0.2
	Galactose	1.2 ± 0.1	1.1 ± 0.3	1.0 ± 0.1	1.1 ± 0.0	1.2 ± 0.2	1.1 ± 0.1	0.8 ± 0.2
	Glucose	46.1 ± 3.8	45.2 ± 2.8	44.8 ± 2.9	44.3 ± 1.5	45.6 ± 3.4	44.8 ± 1.9	46.1 ± 2.8
	Total	56.9 ± 5.1	56.5 ± 3.7	56.5 ± 3.9	54.9 ± 2.4	57.2 ± 4.9	55.9 ± 3.1	56.8 ± 4.8

*Average values were calculated from triplicate (n = 3) of each CB and VB transgenic lines and WT plants*.

### Transgenic Plants Showed Faster Development of the Stem and Roots, Elevation of IAA and Cytokinin Levels, and Upregulation of WUS

**Figure 4:**
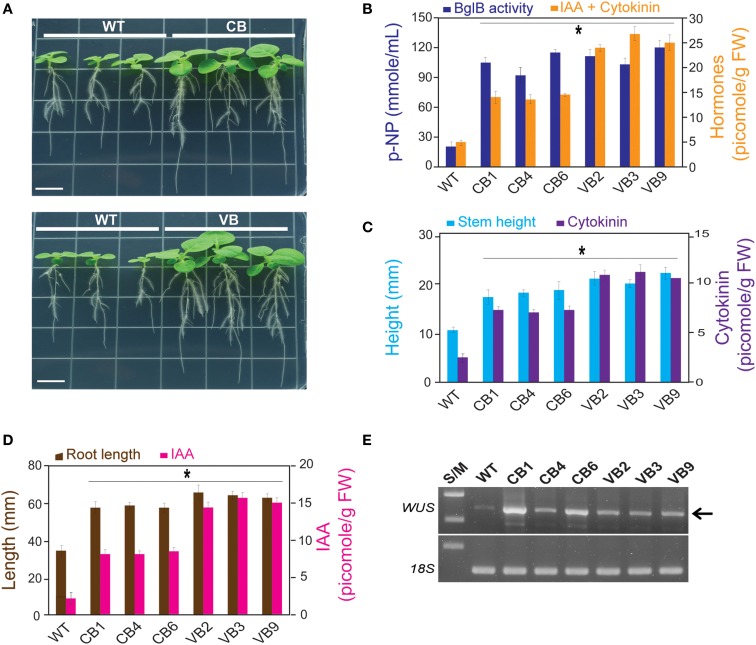
**IAA and cytokinin are involved in faster development of the stem and root of the transgenic plants**. After 15 DAG: **(A)** faster development of the stem and roots of young CB and VB transgenic young plants compared to WT plants; **(B)** Combination of β-glucosidase enzymatic activity and total levels of IAA and cytokinin of the CB and VB transgenic and WT plants; **(C)** Combination of the stem height and cytokinin levels of the transgenic and WT plants; **(D)** Combination of the root lengths and IAA levels of the transgenic and WT plants; **(E)** Transcript expression levels of *N. tabacum WUS* (JQ686923.1) in the transgenic and WT plants. Average values were calculated from triplicate (*n* = 3) of each transgenic lines and WT plants for **(B)**, and twenty individuals (*n* = 20) of each transgenic lines and WT plants for **(C,D)**. *indicates significant differences from the control (WT) (*P* < 0.05).

Based on the increase in stem height and enhanced roots system development, which appeared to be correlated with increased hormone levels of the transgenic plants, we asked whether the increased levels of cytokinin and auxin would affect the development of stems and roots of transgenic seedlings. Seeds from the T_1_ to T_3_ generations, and the WT plants, were sprayed with MS media containing kanamycin. Immediately after the seeds germinated, tiny seedlings were transferred to new MS media in a line to compare stem development. Faster development of the transgenic plants was clearly observed, as presented by the larger size of the transgenic plants compared to WT plants (Figure 4A). At 15 DAG, along with the increase in β-glucosidase enzymatic activity, IAA and cytokinin levels were significantly higher in the transgenic plants compared to WT plants, with the highest hormone level obtained in VB transgenic plants (increase in 585% in VB3 compared to WT plants), whereas there was no difference in β-glucosidase enzymatic activity for the CB and VB transgenic plants (Figure 4B). These results indicate that more liberated hormones were released from the vacuole in VB than in CB transgenic plants.

Correlation analysis between the development of the stem (height) and root system (root lengths) to cytokinin and auxin levels showed a maximum increase in 200% for stem height corresponded to an increase in 458% in cytokinin level in VB9 compared to WT plants (Figure 4C). The maximum increases in roots lengths and IAA level were 186 and 725%, respectively, in VB3 compared to WT plants (Figure 4D). Moreover, these results indicate that, despite slight differences in stem height and root lengths between the CB and VB transgenic plants, the increase in liberated IAA and cytokinin levels promoted faster development in the transgenic lines compared to WT plants. We observed maximum increases in stem height and root lengths of 164 vs. 200%, and 183 vs. 194%, for CB and VB vs. WT plants, respectively. Faster development of the stem and roots was also observed in the T_2_ and T_3_ generations (Figures S3A,B in Supplementary Material). Furthermore, a higher number of leaves and lateral roots, and greater average fresh weight of 20 young plants, were also observed in the transgenic plants compared to WT plants (Figures S3C–E in Supplementary Material), indicating that the faster development of the transgenic plants, compared to WT plants, was stable after three generations.

Plant stem cells are harbored inside the meristem, which is located in the growing tips of the shoots and roots. The faster development observed in the transgenic plants suggests stronger stimulation of stem cell reproduction, which could then induce changes in plant growth and organogenesis (Murray et al., 2012). The population of stem cells in shoot apical meristems is regulated by expression of the homeodomain gene *WUS*, a transcription factor that can be upregulated by cytokinin level. In the root apical meristem, a group of dividing cells, called the quiescent center (QC) in the root apical meristem, is upregulated by IAA level (Yadav et al., 2010; Zhao et al., 2010). To determine the expression levels of *WUS* for transgenic lines and WT plants, RNA was extracted from the stems of young plants (15 DAG) for cDNA synthesis and RT-PCR. The results showed higher *WUS* expression levels in the transgenic plants compared to WT plants (Figure 4E), providing evidence that superior development of the transgenic plants compared to WT plants was due to elevated hormone levels.

### Enhanced Resistance to NaCl Stress and Elevation of ABA in Transgenic Plants

**Figure 5:**
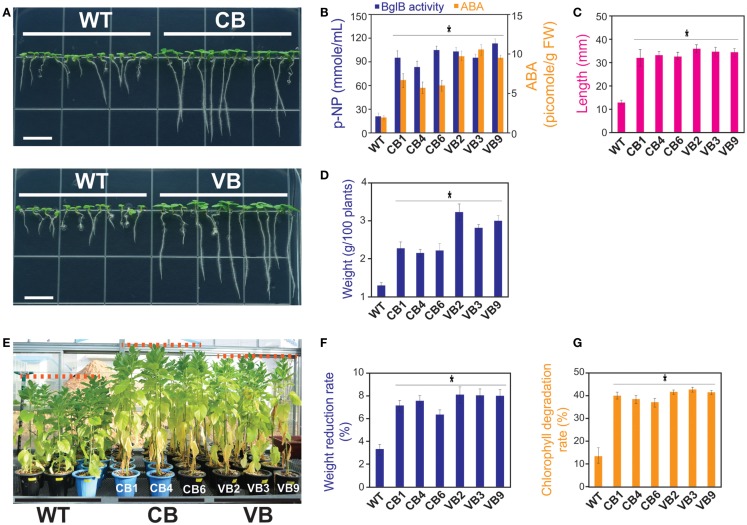
**Heterologously expressed BglB transgenic plants showed higher tolerance to salt stress in young seedlings, but triggered programed cell deaths occurred sooner in mature plants compared to WT plants**. After 15 DAG in MS media containing 200 mM NaCl: **(A)** Faster development of the stems and roots of the CB and VB transgenic seedlings; **(B)** Combination of β-glucosidase enzymatic activity and ABA levels of the transgenic and WT seedlings; **(C)** Root lengths; and **(D)** Fresh weight of 100 seedlings of the transgenic and WT seedlings. Average values were calculated from triplicated. After 10 days treated with 200 mM NaCl in the greenhouse: **(E)** More senescent leaves appeared in the transgenic plants compared to WT plants; **(F)** Weight reduction rate; and **(G)** Chlorophyll degradation rate of the transgenic and WT plants. Average values were calculated from triplicate (*n* = 3) of each transgenic lines and WT plants for **(B)**, twenty seedlings (*n* = 20) of each transgenic lines and WT plants for **(C)**, triplicate (*n* = 3) of each transgenic lines and WT plants for **(D)**, ten individuals (*n* = 10) of each transgenic lines and WT plants for **(F,G)**. *indicates significant differences from the control (WT) (*P* < 0.05).

Next, we asked whether increased ABA levels in the transgenic plants led to increased tolerance of salt stress, as mentioned in previous studies (Lee et al., 2006; Wang et al., 2011; Han et al., 2012; Xu et al., 2012). Seeds from the T_1_ to T_3_ generations were used, and after germination, tiny seedlings were transferred to new MS media containing 200 mM NaCl to examine the response to high NaCl stress. Observations at 15 DAG showed that the transgenic plants were more resistant to high NaCl, as indicated by enhanced development in the transgenic compared to WT seedlings in term of increased root length, number of leaves, number of lateral roots, and fresh weight (Figure 5A). As shown in Figure 5B, the higher ABA level was clearly related to increased β-glucosidase enzymatic activity in the transgenic seedlings, with the highest ABA levels recorded in the VB transformants (maximum increase in 504% in VB3 compared to WT seedlings). Increased tolerance to high NaCl stress was also displayed by the obviously longer root lengths and greater fresh weight of 100 transgenic seedlings compared to WT seedlings (maximum increase in 271% in root lengths and 256% in fresh weight in the VB2 compared to WT seedlings; Figures 5C,D).

Next, to examine the resistance of mature plants to salt stress, transgenic and WT plants grown in the greenhouse were subjected to a salt stress experiment at 80 DAG. As shown in Figure 5E, more senescent leaves appeared in the transgenic than WT plants, which may explain the higher rate of weight reduction (8.3% in VB2 compared to 3.4% in WT plants; Figure 5F). The appearance of leaves senescence indicated that a faster programed cell death process occurred in the transgenic than WT plants, which was also represented by the higher rate of chlorophyll degradation in transgenic plants (43.2% in VB3 compared to 14.1% in WT plants; Figure 5G).

## Discussion

Because of its thermostability and transglycosylation properties, the *T. maritima* BglB enzyme is considered to be a useful catalyst for biotechnological applications (Goyal et al., 2001). According to Jung et al. (2010), transgenic tobacco plants can not only be utilized for the mass production of BglB, but also, the overexpression of heterologous BglB in tobacco has led to changes in phenotypic characteristics (such as larger leaves and taller plants) (Jung et al., 2013). Plants contain their own β-glucosidase genes, and previous studies have demonstrated that the expression of β-glucosidase, including heterologous expression, affects the hydrolysis of hormone conjugates and homeostasis in plants, which in turn control plant development (Schliemann, 1984; Brzobohaty et al., 1993; Dietz et al., 2000; Kiran et al., 2006). In the present study, by observing pronounced phenotypic changes in the *T. maritima* BglB transgenic tobacco compared to WT plants. The transgenic tobacco remained stable over three offspring generations (Figures 3–5). We were encouraged to evaluate the relationship between β-glucosidase enzymatic activity of *T. maritima* BglB and changes in plant hormone levels.

For vacuole targeting, among the three different types of vacuolar sorting signals (N- or C-terminal polypeptides or internal sequences) that have been identified (Jiang and Rogers, 1998; Matsuoka and Neuhaus, 1999), C-terminal polypeptides, such as the C-terminal amino acids AFVY tetrapeptide from phaseolin, are considered be the most efficient (Frigerio et al., 2001; Nausch et al., 2012a,b). However, due to the presence of numerous hydrolytic enzymes in the vacuole of plant cells, it is generally difficult for proteins to maintain their activity inside the vacuole (Boller and Kende, 1979; Marty, 1999). Here, we showed that the β-glucosidase enzymatic activity of heterologously expressed BglB was significantly higher in the transgenic (both the CB and VB transformants) plant compared to WT plants (Figure 2A). The transgenic plants remained stable during the life cycle and durable after three offspring generation (Figures 2A,B). These results indicate that the *T. maritima* BglB was effectively expressed in the transgenic tobacco plants.

For the first time, the present of the heterologous BglB and β-glucosidase enzymatic activity assays were conducted after vacuole isolation, which showed that BglB expression was dramatically higher in the VB than CB transgenic plants (Figures 1E and 2C). This result clearly indicated that AFVY tetrapeptide were effective for sorting *T. maritima* BglB into the vacuole, and that its β-glucosidase enzymatic activity was maintained and could tolerate the protein-degrading conditions of the vacuole environment. Therefore, vacuole-targeted *T. maritima* BglB transgenic plants should be considered candidates for plant molecular farming, where plants are used as bioreactors to produce degrading enzymes for hydrolysis of lignocellulosic material, which is similar to chloroplast-targeted *T. maritima* BglB transgenic plants (Jung et al., 2010, 2013).

Hormone glucoside conjugates, which are mainly stored in the plant vacuole, are considered inactive forms in hormone metabolism, and can be liberated by β-glucosidases, a large group of enzymes that can hydrolyze glucoside ester linkages (Sembdner et al., 1994; Bajguz and Piotrowska, 2009). A wide variety of β-glucosidase enzymes from plants have been proven to be hormone conjugates with hydrolysis capability (Schliemann, 1984; Brzobohaty et al., 1993; Dietz et al., 2000; Kiran et al., 2006; Lee et al., 2006; Yao et al., 2007; Jin et al., 2011). We demonstrated a novel approach in which transformation of BglB, encoding a thermostable β-glucosidase from the bacterium *T. maritima* (Goyal et al., 2001), affected plant hormone levels through hydrolyzation of glucoside ester links in hormone conjugates in the transgenic plants, which seemed to be the result of non-specific activity. For example, previous studies demonstrated that each kind of β-glucosidase likely performs its functions in specific hormone conjugates (Brzobohaty et al., 1993; Dietz et al., 2000). Kiran et al. (2012) reported that Zm-p60.1 is capable of releasing active cytokinin from O- and N-glucosides, and confirmed that the liberated hormones are still in the active state. Knowledge of the transportation mechanism from inside to outside of the vacuole is still lacking (Vitale and Hinz, 2005; De Marcos Lousa et al., 2012). In the present study, significantly higher enzymatic activity, particularly in isolated vacuoles, was accompanied by dramatically higher levels of hormones (IAA, ABA, and cytokinin) in the VB plants compared to CB transgenic plants, with WT plants showing the lowest levels (Figure 2C). These results clearly demonstrated that, when greater amounts of BglB were targeted to the vacuole, more liberated hormones were released.

In contrast to the results obtained by Kiran et al. (2012), who found no significant phenotypic changes in vacuole-targeted *Zm-p60.VAL* transgenic plants, our results showed pronounced phenotypic changes in *T. maritima* BglB transgenic plants compared to WT plants. Mature transgenic plants exhibited enhanced development, in terms of faster growth in stem height and a shortened growth cycle, with earlier flowering (Figure 3). Young seedlings had increased stem height and longer roots (Figure 4), which were accompanied by significantly higher hormones levels that were maintained over three offspring generations of the transgenic plants. These results provide clear evidence that heterologously expressed BglB increases the plant hormones levels, which then influence their phenotypes.

Due to the elevated levels of IAA, ABA, and cytokinin, it is difficult to determine the specific factor that directly contributes to the phenotypic changes in the transgenic plants. Fortunately, previous works can provide clues to trace the cause of such changes. For example, IAA is known to regulate root development (Overvoorde et al., 2010), cytokinin plays pivotal roles in the formation and activity of shoot meristems (Werner et al., 2003; Werner and Schmülling, 2009), and ABA functions in the plant response to dehydrating/salinity stresses and programed cell death (Finkelstein, 2006; Yang et al., 2014). Previous studies have also shown that the reproduction and differentiation of stem cells harbored in the shoot and root apical meristem contribute to development and organogenesis in plants (Williams and Fletcher, 2005; Powell and Lenhard, 2012). Therefore, the taller stem height, longer roots, and earlier flowering observed in the transgenic plants could indicate enhancement of the shoot and root apical meristem in the transgenic plants compared to WT plants. Specifically, the expression level of *WUS*, a transcription factor that regulates the development and division of stem cells in the shoot apical meristem, is upregulated by cytokinin (Kurakawa et al., 2007; Werner and Schmülling, 2009; Zhao et al., 2010), shedding light on the mechanism contributing to the role of cytokinin, which was increased in our transgenic plants, in enhancing the development of the stems and aboveground organs.

Our result showed enhanced development of the root systems (represented by increased roots dry weight, number of lateral roots, and root length), confirming the effect of a larger amount of IAA on the development of root systems in the transgenic plants (Figures 3 and 4). ABA mainly functions in the plant’s response to dehydration by inducing stomatal opening/closing, and also plays a role in limiting cell division and expansion, decreasing shoot growth and lateral root initiation, and promoting developmental phase changes such as vegetative-to-reproductive transitions (Finkelstein, 2006). In the present study, the increased ABA levels were related to increased salt stress tolerance in young seedlings. The faster chlorophyll degradation and higher rates of weight reduction after treatment with NaCl solution in mature plants revealed that programed cell death was promptly triggered in the transgenic plants for both the VB and CB transformants, compared to WT plants (Figure 5). Notably, no significant difference in β-glucosidase enzymatic activity, but significantly higher hormones levels, in the VB transgenic plants compared to CB transgenic plants, were observed, confirming that the hormone conjugates are mainly stored in the vacuole, and more liberated hormones were released from the conjugates in the VB transgenic plants, which contributed to the greater effect on plant development in the VB transgenic plants.

## Conclusion

After *T. maritima BglB* was first overexpressed and effectively targeted into the vacuole by the addition of AFVY C-terminal tetrapeptides, BglB was still active and functional. The main results emerging from this study are that the hormone (ABA, IAA, and cytokinin) conjugates are mainly stored in the vacuole, and perhaps more importantly, higher levels of hormones liberated from their conjugates via BglB-mediated hydrolysis enhance the growth and development in VB transgenic plants to a greater extent than in CB transgenic plants. Therefore, the use of heterologously overexpressed vacuole-targeted *T. maritima BglB* may be an approach to develop molecular farming technology to achieve multiple targets: increased production of the β-glucosidase BglB, increased biomass accumulation, and shortened of developmental stages. Also this *BglB* vacuole-targeted plant farming system influences of total biomass accumulation and as such may be useful in increasing biomass production for bioenergy and biofuel production.

## Conflict of Interest Statement

The authors declare that the research was conducted in the absence of any commercial or financial relationships that could be construed as a potential conflict of interest.
