# Supplementary material for: Phenotypic Changes in Transgenic Tobacco Plants Overexpressing Vacuole-Targeted Thermotoga maritima BglB Related to Elevated Levels of Liberated Hormones
Source: Front Bioeng Biotechnol. 2015 Nov 9;3:181. doi: 10.3389/fbioe.2015.00181 (PMC4642495; doi:10.3389/fbioe.2015.00181)
Supplement: Supplementary file 1 [file Presentation_1.PDF]

Phenotypic changes in transgenic tobacco plants overexpressing vacuole-targeted *Thermotoga maritima* BglB related to elevated levels of liberated hormones

Quynh Anh Nguyen[1], Dae-Seok Lee[2], Jakyun Jung[1], Hyeun-Jong Bae[1,2,*]

Supplementary data

Supplementary Figures:

Supplementary Figure S1. Quantitative of IAA, ABA, and cytokinin at 14 DAG.

Supplementary Figure S2. (**A**) Dry weight of stem, leaves, and roots of WT and transgenic plants (T_1 generation) after seed harvest. (**B**) Time from germination to flowering of the T_2 and T_3 generations of transgenic plants and WT plants. (**C**) Hormone levels at flowering of the transgenic and WT plants. (**D**) Stem height and (**E**) Dry weight of the T_2 and T_3 generations of transgenic plants and WT plants.

Supplementary Figure S3. (**A**) Stem height and (**B**) Root lengths of the T_2 and T_3 generation of transgenic plants and WT plants. (**C**) Number of leaves; (**D**) Number of lateral roots; and (**E**) Fresh weight of 20 plants of the transgenic and WT plants.

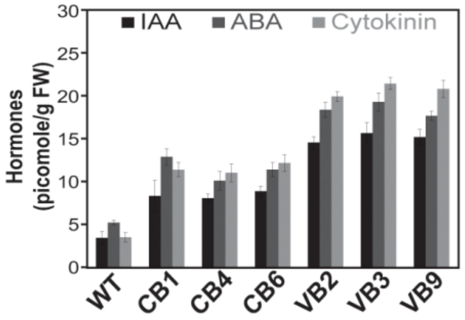

Supplementary Figure S1. Quantitative of IAA, ABA, and cytokinin at 14 DAG.

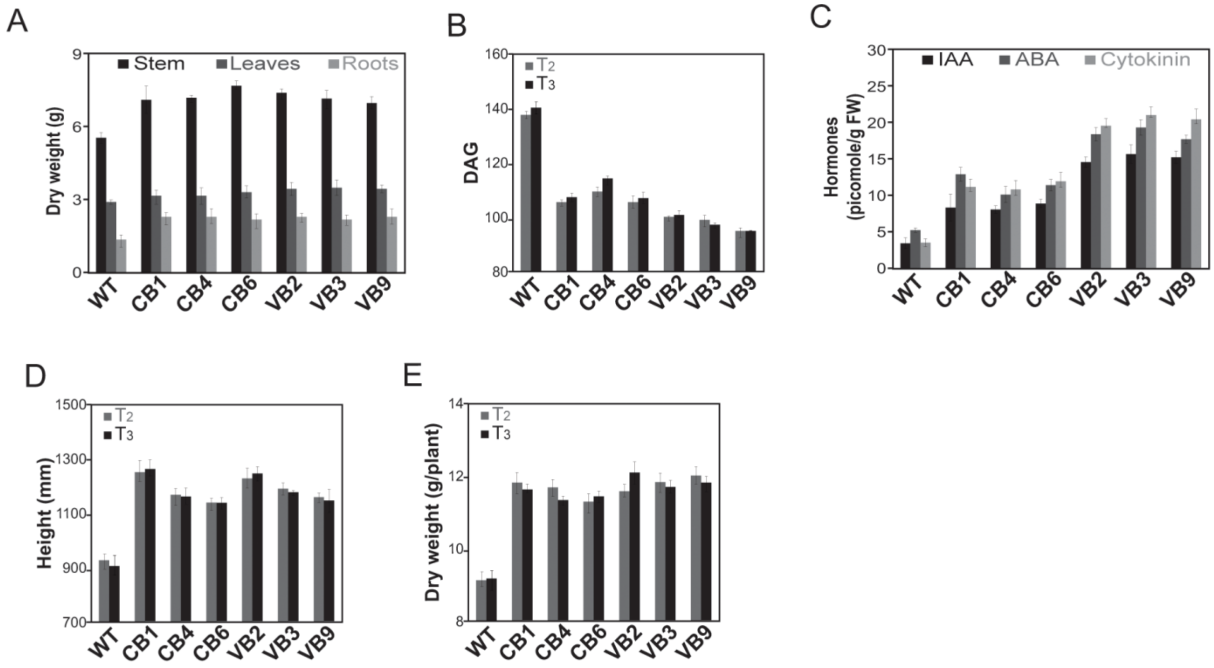

Supplementary Figure S2. (**A**) Dry weight of stem, leaves, and roots of WT and transgenic plants (T_1 generation) after seed harvest. (**B**) Time from germination to flowering of the T_2 and T_3 generations of transgenic plants and WT plants. (**C**) Hormone levels at flowering of the transgenic and WT plants. (**D**) Stem height and (**E**) Dry weight of the T_2 and T_3 generations of transgenic plants and WT plants.

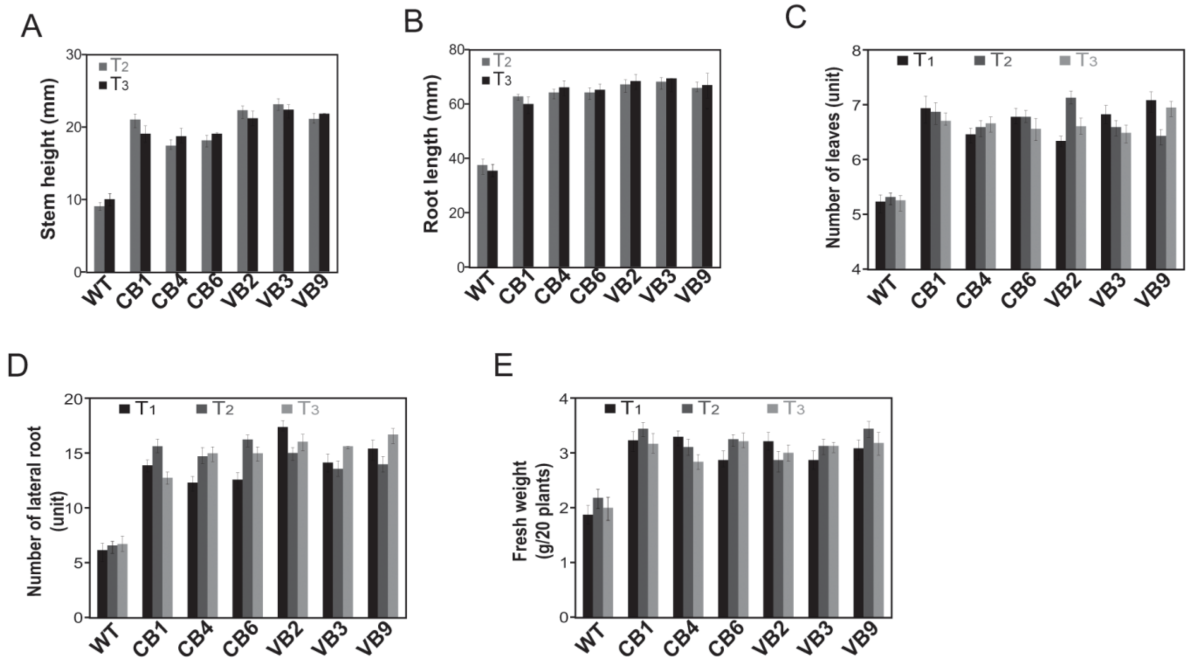

Supplementary Figure S3. (**A**) Stem height and (**B**) Root lengths of the T_2 and T_3 generation of transgenic plants and WT plants. (**C**) Number of leaves; (**D**) Number of lateral roots; and (**E**) Fresh weight of 20 plants of the transgenic and WT plants.
